# Oral *Lactobacillus* strains reduce cytotoxicity and cytokine release from peripheral blood mononuclear cells exposed to *Aggregatibacter actinomycetemcomitans* subtypes in vitro

**DOI:** 10.1186/s12866-020-01959-5

**Published:** 2020-09-11

**Authors:** Nuntiya Pahumunto, Amina Basic, Anna-Karin Östberg, Rawee Teanpaisan, Gunnar Dahlen

**Affiliations:** 1 Common Oral Diseases and Epidemiology Research Center and Department of Stomatology, Faculty of Dentistry, Prince of Songkla University, Hat Yai, Thailand; 2 Department of Oral Microbiology and Immunology, Institute of Odontology, Sahlgrenska Academy, University of Gothenburg, Gothenburg, Sweden

**Keywords:** Microbial interaction, Oral lactobacilli, *Aggregatibacter actinomycetemcomitans*, Bacterial toxicity, Cytokine release, Peripheral blood mononuclear cells

## Abstract

**Background:**

This study evaluated the effect of oral lactobacilli on the cytotoxicity and cytokine release from peripheral blood mononuclear cells (PBMCs) when exposed to *Aggregatibacter actinomycetemcomitans* subtypes in vitro*.* The supernatants and cell wall extracts (CWEs) of eight *A. actinomycetemcomitans* strains, representing different subtypes, and three *Lactobacillus* strains were used. The PBMCs from six blood donors were exposed to supernatants and CWEs of *A. actinomycetemcomitans* or *Lactobacillus* strains alone or combinations and untreated cells as control. The cytotoxicity was determined by trypan blue exclusion method and IL-1β secretion by ELISA. TNF-α, IL-6, and IL-8 secretions were measured using Bioplex Multiplex Immunoassay.

**Results:**

Supernatants or CWEs from all bacterial strains showed cytotoxicity and IL-1β secretion and the subtypes of *A. actinomycetemcomitans* showed generally a significantly higher effect on PBMCs than that of the *Lactobacillus* strains. Two highly toxic *A. actinomycetemcomitans* strains (JP2 and JP2-like) induced a higher response than all other strains. When combined, *Lactobacillus* significantly reduced the toxicity and the IL-1β secretion induced by *A. acinomycetemcomitans*. The effect varied between the subtypes and the reduction was highest for the JP2 and JP2-like strains. The *Lactobacillus paracasei* strain SD1 had a higher reducing effect than the other *Lactobacillus* strains. This strain had a consistent reducing effect on all subtypes of *A. actinomycetemcomitans* cytotoxicity, and release of IL-1β, IL-6, IL-8, and TNF-α from PBMCs of the blood donors. A strong and significant variation in cytokine release between the six blood donors was noticed.

**Conclusions:**

*Lactobacillus* spp. and *L. paracasei* SD1 in particular, showed a limited but statistically significant reducing interaction with *A. actinomycetemcomitans* toxicity and release of cytokines in vitro.

## Background

Periodontitis is an inflammatory disease induced by the dental biofilm and affecting the tooth supporting tissues, bone and connective tissues, which may result in tooth loss. Several bacterial species have been associated with periodontitis and been designated major periodontopathogens [1]. *Aggregatibacter actinomycetemcomitans* has been associated with periodontitis in both young and older individuals [2, 3]. *A. actinomycetemcomitans* produces a leukotoxin (Ltx), an exotoxin targeting cells of the immune system in the periodontal tissues [4]. Leukotoxin induces a pro-inflammatory response by activation of macrophages/monocytes and secretions of IL-1β, and also selectively kills human leukocytes [5]. The leukotoxic activity among *A. actinomycetemcomitans* strains and subtypes is variable, and a highly toxic clone has been identified [6]. This genotype, termed the JP2 clone, primarily found in North and West African populations, has been closely associated with periodontitis in young individuals [6, 7]. Previous studies on various subtypes of *A. actinomycetemcomitans* strains isolated from an adult Thai population with periodontitis, revealed a significant variation in IL-8 cytokine expression (but not IL-1β, IL-6, and TNF-α) in human gingival epithelial cells (HGECs) [8, 9]. Non-serotypable strains (NS1 and NS2) and a JP2-like strain showed significantly lower IL-8 responses than the serotypable (serotype a-f) stains [9].

In the complex dental biofilm numerous interactions take place in order to regulate the microbial community by synergistic and antagonistic forces and metabolic networks [10]. Oral streptococci, which are abundant in most individuals, are considered to play a major role in regulating the dental biofilm ecology by producing interfering metabolic products and bacteriocins against a number of anaerobic gram-negative bacteria commonly associated with periodontitis including *A. actinomycetemcomitans* [11, 12]. Similarly, *Lactobacillus* species have shown inhibitory activities on the toxic effect of *A. actinomycetemcomitans* [13]. The purpose of the present study was to investigate the factors that are involved in the bacterial interaction between beneficial bacteria such as lactobacilli and a periodontopathogen such as *A. actinomycetemcomitans*. More specifically, the aim of this study was to examine the potential inhibiting or reducing effect of oral *Lactobacillus* strains on the cytotoxicity and secretion of the cytokines IL-1β, IL-6, IL-8 and TNF-α in peripheral blood mononuclear cells (PBMCs) by cell wall extracts (CWEs) and supernatants from a spectrum of *A. actinomycetemcomitans* subtypes.

## Results

### Cytotoxicity and IL-1β release from PBMCs by *A. actinomycetemcomitans* subtypes and *Lactobacillus* spp.

**Fig. 1:**
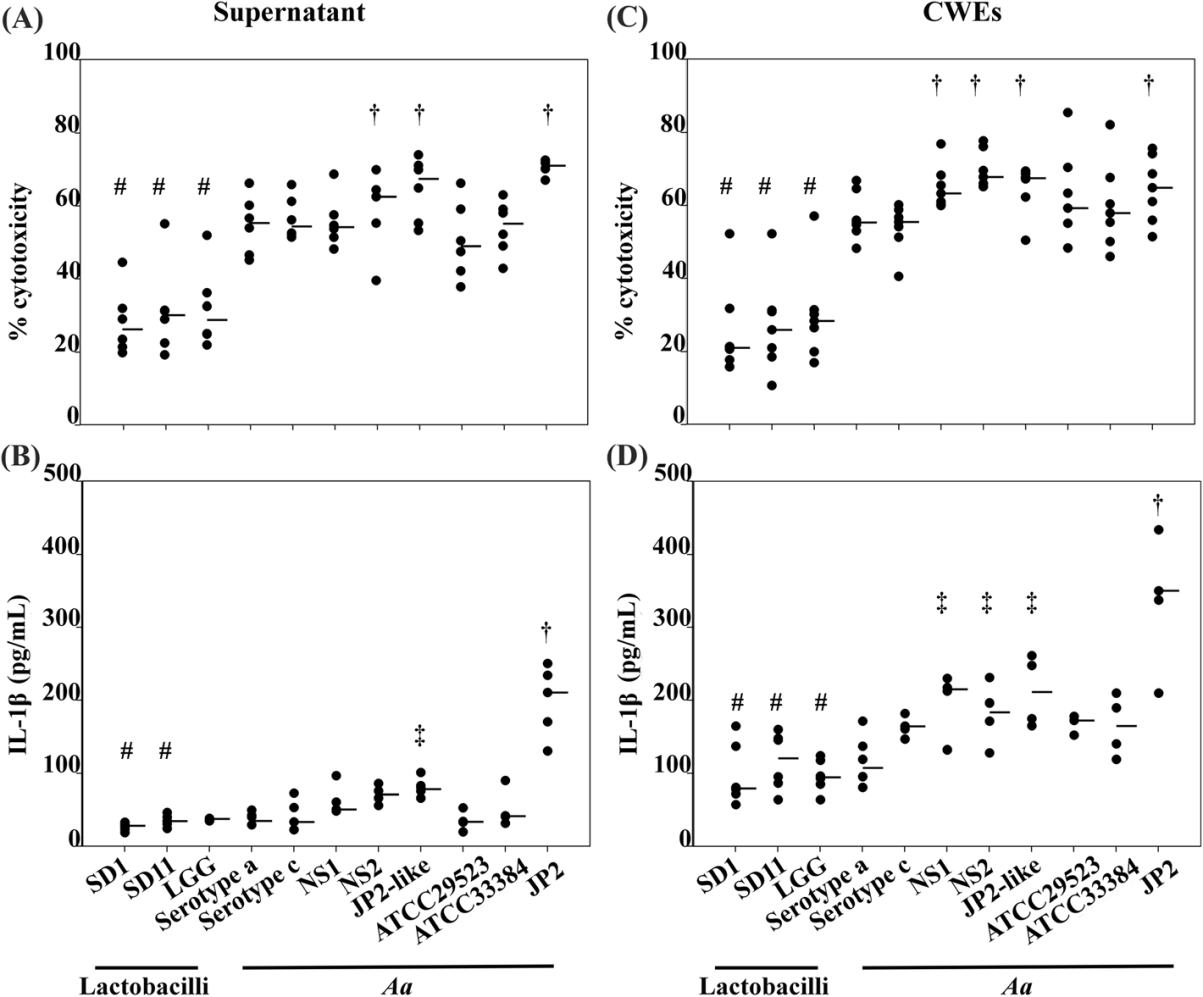
The cytotoxicity (**a** and **c**) and IL-1β secretion (**b** and **d**) of the PBMCs exposed to the supernatant and CWEs of lactobacilli and *A. actinomycetemcomtians*, respectively. ^#^Statistically significant lower cytotoxicity (*p* < 0.001) or IL-1β secretion (*p* < 0.042) of lactobacilli compared to *A. actinomycetemcomtians*, ^†^Statistically significant higher cytotoxicity of *A. actinomycetemcomtians* strain NS1, NS2, JP2-like, and OMG3952 (*p* < 0.001) and IL-1β secretion of *A. actinomycetemcomtians* strain OMG3952 compared to other strains (*p* < 0.001), ‡ Statistically significant higher IL-1β secretion of *A. actinomycetemcomtians* strain NS1, NS2 and JP2-like, except OMG3952, compared to other strains (*p* < 0.045). The statistical method was Mann Whitney U test and the median of the group is shown as a horizontal line

The cytotoxicity and IL-1β release upon stimulation with supernatant and CWEs of individual strains of lactobacilli and *A. actinomycetemcomitans* strains are shown in Fig. 1. Treatment of PBMCs with supernatants from the three lactobacilli strains resulted in lower cytotoxicity, approximately 1.2–2.5 folds (ranged 25.6 ± 4.8 to 28.7 ± 5.6%), and IL-1β secretion, approximately 1.5–3.3 folds (ranged 26.7 ± 5.2 to 36.9 ± 1.3 pg/mL), compared to when treated with *A. actinomycetemcomitans* strains NS1, NS2, JP2-like, and JP2 clone (*p* < 0.05, Fig. 1a and b). Also, NS2, JP2-like, and JP2 clone showed significantly higher cytotoxicity and IL-1β secretion compared to serotype a, serotype c, NS1, ATCC33384 (serotype c) and ATCC29523(serotype a) (*p* < 0.05, Fig. 1a and b). The significance for the higher release of IL-1β by the JP2 clone (OMG3952) in comparison with ATCC 33384 and ATCC 29523 was *p* < 0.041 and *p* < 0.026 respectively.

The percentage of cytotoxicity and IL-1β secretion induced by CWEs from *A. actinomycetemcomitans* strains was higher than lactobacilli strains, approximately 1.4–3.4 folds (ranged 50.4 ± 7.4 to 70.6 ± 2.1%) and 1.4–3.1 folds (ranged 31.7 ± 13.6 to 194.3 ± 45.4 pg/mL), respectively. Also, NS1, NS2, JP2-like, and JP2 clone showed significantly higher cytotoxicity and IL-1β secretion than serotype a, serotype c, ATCC33384 (serotype c) and ATCC29523 (serotype a) (*p* < 0.05, Fig. 1c and d).

The results of PBMCs treated with CWEs of tested strains were in line with the outcome of supernatant treatment by that *A. actinomycetemcomitans* strains and showed significantly greater cytotoxicity and IL-1β induction compared to lactobacilli strains (Fig. 1c and d).

### The effect of *Lactobacillus* spp. on the cytotoxicity and IL-1β release from PBMCs by *A. actinomycetemcomitans*

**Fig. 2:**
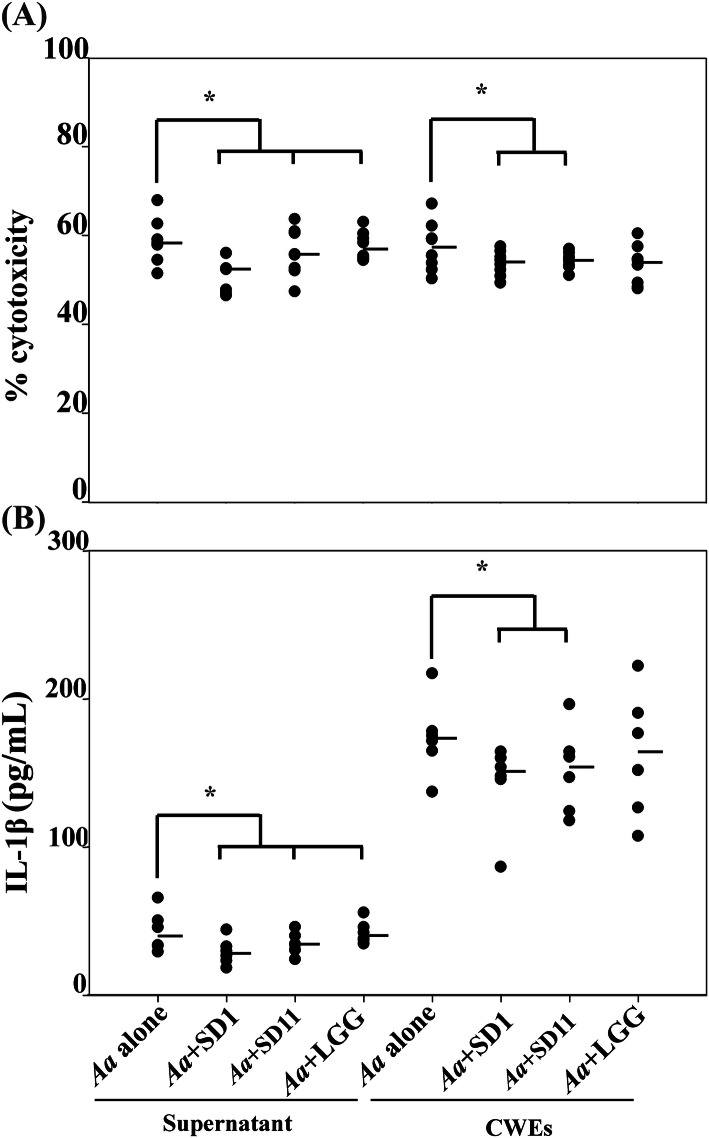
The cytotoxicity (**a**) and IL-1β concentration (**b**) secreted from PBMCs (from six blood donors) exposed to the supernatant (1:1 concentration) or CWEs (100 μg/mL), where each spot represents one blood donor and a mean value of eight *A. actinomycetemcomitans* strains alone or in combination with three strains of *Lactobacillus* spp. (SD1, SD11 or LGG). * Statistically significant lower cytotoxicity (*p* < 0.001) or IL-1β concentration (*p* < 0.048) for the combinations compared to *A. actinomycetemcomitans* alone using Mann Whitney U test. The median of the group is shown as a horizontal line

The supernatant of *Lactobacillus* strains showed a varied reducing capacity on *A. actinomycetemcomitans* induced cytotoxicity and IL-1β release on PMBCs from six blood donors. *L. paracasei* SD1 reduced both cytotoxicity and IL-1β release from PBMCs by *A. actinomycetemcomitans* strains (mean value of 8 strains) significantly approximately 0.5–1.3 folds (from 58.9 ± 4.7 to 51.5 ± 3.7% and 0.7–1.2 folds (42.9 ± 4.6 to 29.1 ± 3.0 pg/mL; *p* < 0.05; Fig. 2a and b). The CWEs of *L. paracasei* SD1 showed in line with supernatant a 0.8–1.7 fold decreased cytotoxicity (from 57.6 ± 5.3 to 53.9 ± 2.7%, *p* < 0.05; Fig. 2a) and 0.5–1.2 fold reduction of IL-1β secretion (from 174.0 ± 32.8 to 143.1 ± 27.3 pg/mL, *p* < 0.001; Fig. 2b) of *A. actinomycetemcomitans* CWEs. Also, *L. rhamnosus* SD11 reduced significantly IL-1β secretion by *A. actinomycetemcomitans* CWEs 0.7–1.2 fold (from 174.0 ± 32.8 to 151.8 ± 28.9 pg/mL, *p* < 0.001; Fig. 2b). The cytotoxicity and IL-1β secretion did not reach statistical significance for *A. actinomycetemcomitans* CWEs combined with CWEs of LGG (0.6–0.9 fold reduction of cytotoxicity and 0.7–0.9 fold reduction of IL-1β secretion from 174.0 ± 32.8 to 164.5 ± 42.5 pg/mL; *p* > 0.05; Fig. 2a and b).

### Effect of *Lactobacillus paracasei* SD1 on the cytotoxicity and IL-1β release by CWEs from *A. actinomycetemcomitans* subtypes

The cytotoxicity of individual *A. actinomycetemcomitans* strains after combination with *L. paracasei* SD1 was significantly lower compared with *A. actinomycetemcomitans* alone (*p* < 0.01; Fig. 4). The reducing effect of *L. paracasei* SD1 was 1.0–1.6 folds. NS1, NS2 and JP2-like strains revealed significant reduction compared to each of them alone showing 1.0–1.4 folds of reduction (Fig. 4). Individual variations on the response of the PBMCs were however, noticed.

**Fig. 3:**
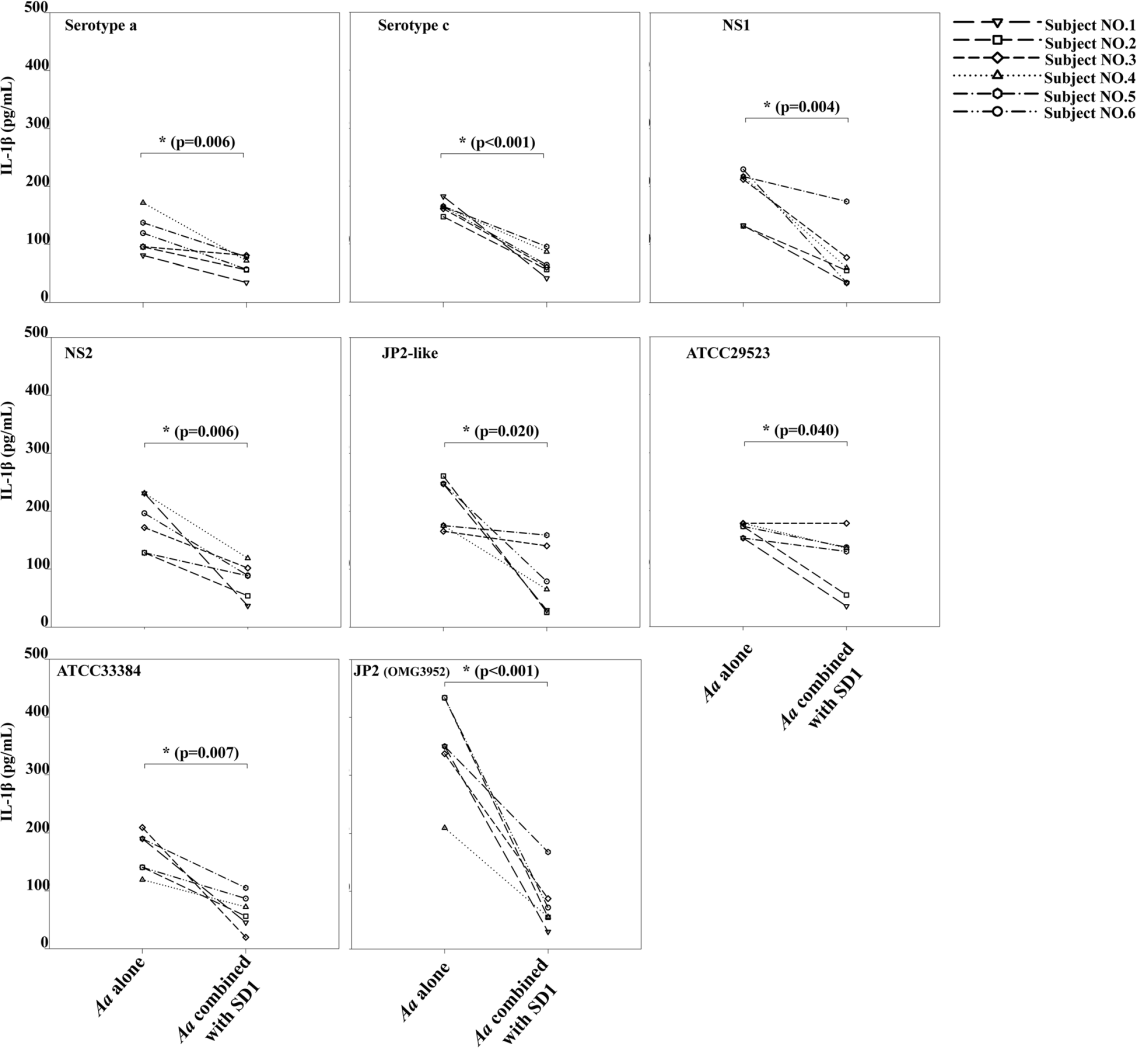
IL-1β concentration secreted from PBMCs from six blood donors exposed to CWEs (100 μg/mL) of each strain of *A. actinomycetemcomitans* and their combination with *Lactobacillus paracasei* SD1. *Statistically significant IL-1β concentration for the combinations compared to *A. actinomycetemcomitans* alone (Wilcoxon Signed-Rank Test; *p* < 0.05)

**Fig. 4:**
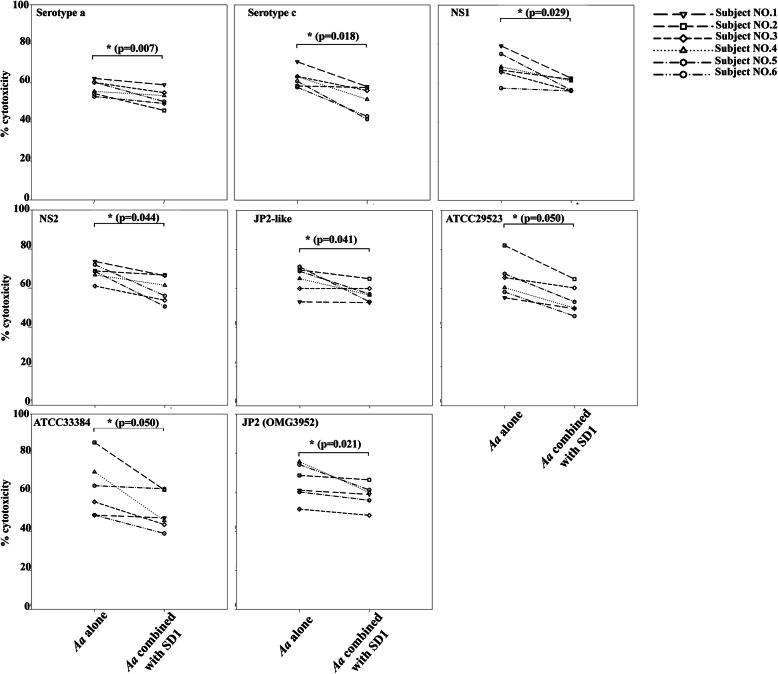
Cytotoxicity of PBMCs from six blood donors exposed to CWEs (100 μg/mL) of each strain of *A. actinomycetemcomitans* and their combination with *Lactobacillus paracasei* SD1. *Statistically significant cytotoxicity for the combinations compared to *A. actinomycetemcomitans* alone (Wilcoxon Signed-Rank Test; *p* < 0.05)

The ability of *L. paracasei* SD1 to reduce cell responses induced by separate *A. actinomycetemcomitans* strains is shown in Fig. 3 and Fig. 4. The CWEs of *L. paracasei* SD1 proved to have a significant and consistent negative impact on IL-1β secretion in all cell cultures treated with the different *A. actinomycetemcomitans* strains (*p* < 0.01). The adding of *L. paracasei* SD1 to the cell cultures reduced the effect of each *A. actinomycetemcomitans* strain approximately 1.0–11.7 folds compared to when the cells were cultured with *A. actinomycetemcomitans* alone (Fig. 3). NS1, NS2, and JP2-like (ranged 128.1 to 261.0 pg/mL) showed a significant reduction approximately 1.2–10.2 folds compared with the addition of *L. paracasei* SD1 (ranged 25.6 to 174.3 pg/mL) in all subjects. Also, here, large individual variations were seen between the PBMCs.

### Effect of *A. actinomycetemcomitans* subtypes alone and combination with *L. paracasei* SD1 on IL-6, IL-8 and TNF-α release from PBMCs

**Fig. 5:**
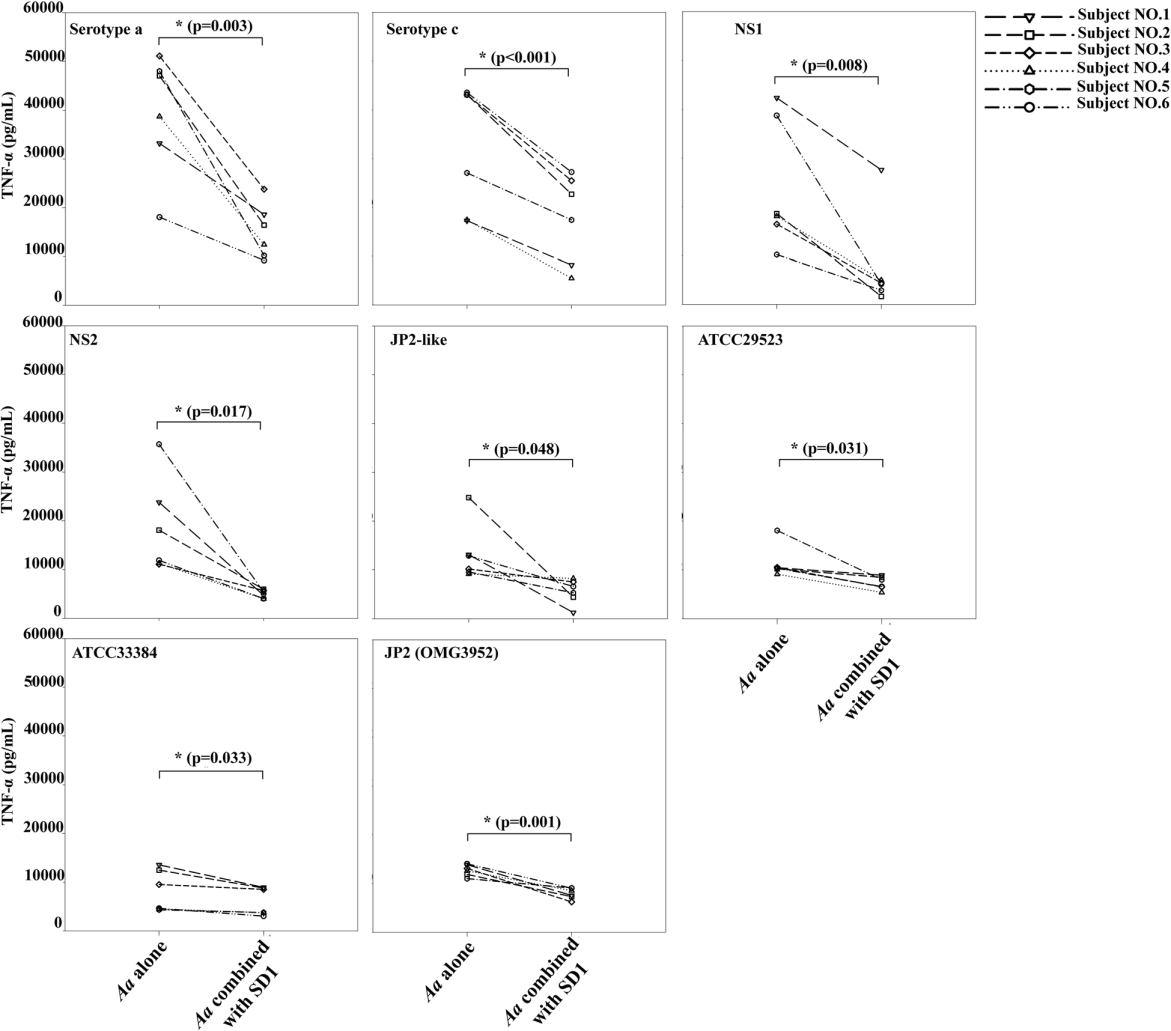
TNF-α secreted from PBMCs from six blood donors and exposed to CWEs (100 μg/mL) of each strain of *A. actinomycetemcomitans* and their combination with *Lactobacillus paracasei* SD1. * Statistically significant cytotoxicity for the combinations compared to *A. actinomycetemcomitans* alone (Wilcoxon Signed-Rank Test; *p* < 0.05)

**Fig. 6:**
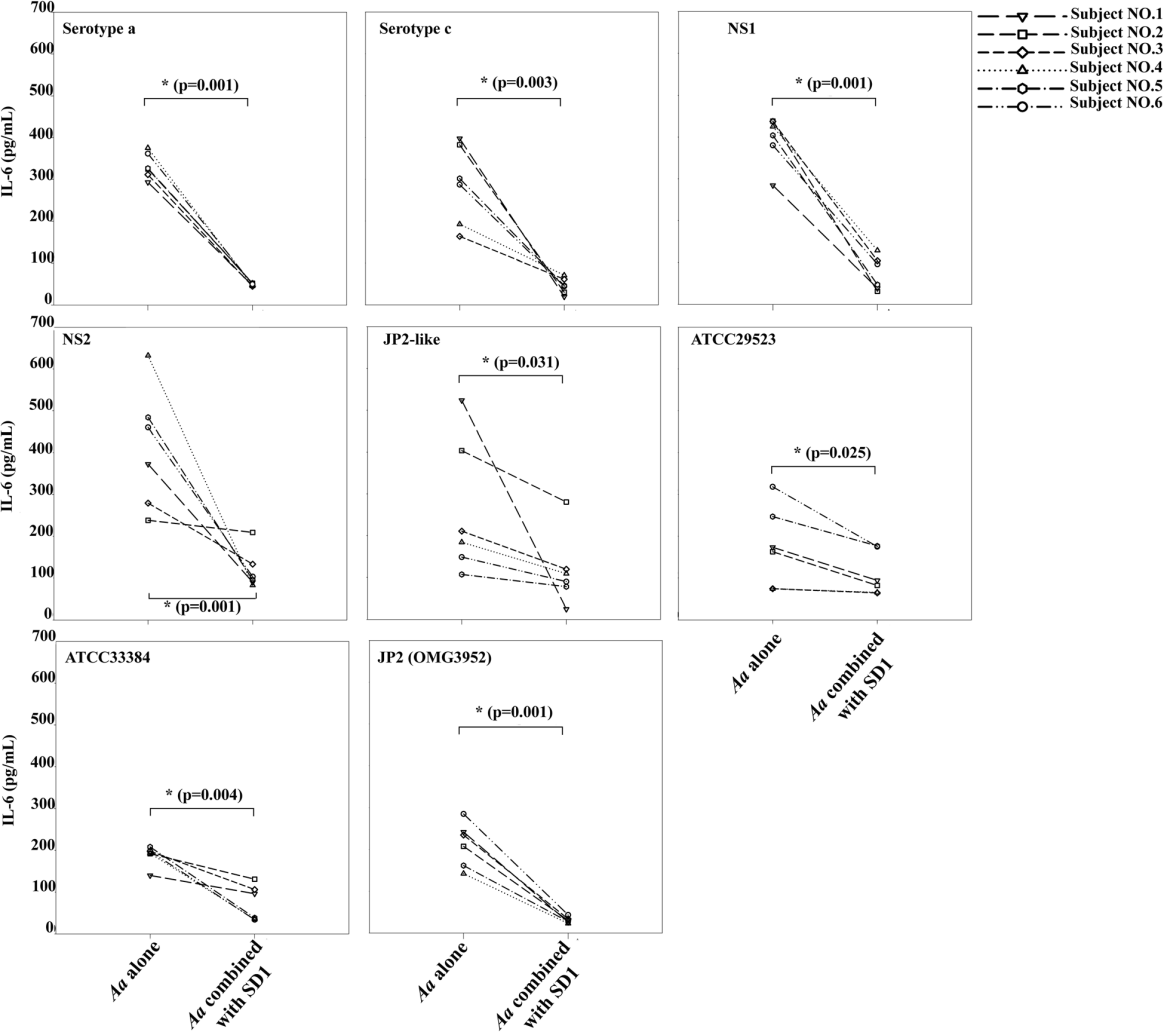
IL-6 secreted from PBMCs from six blood donors and exposed to CWEs (100 μg/mL) of eight strains of *A. actinomycetemcomitans* alone and in combination with *Lactobacillus paracasei* SD1. * Statistically significant cytotoxicity for the combinations compared to *A. actinomycetemcomitans* alone (Wilcoxon Signed-Rank Test; *p* < 0.05)

**Fig. 7:**
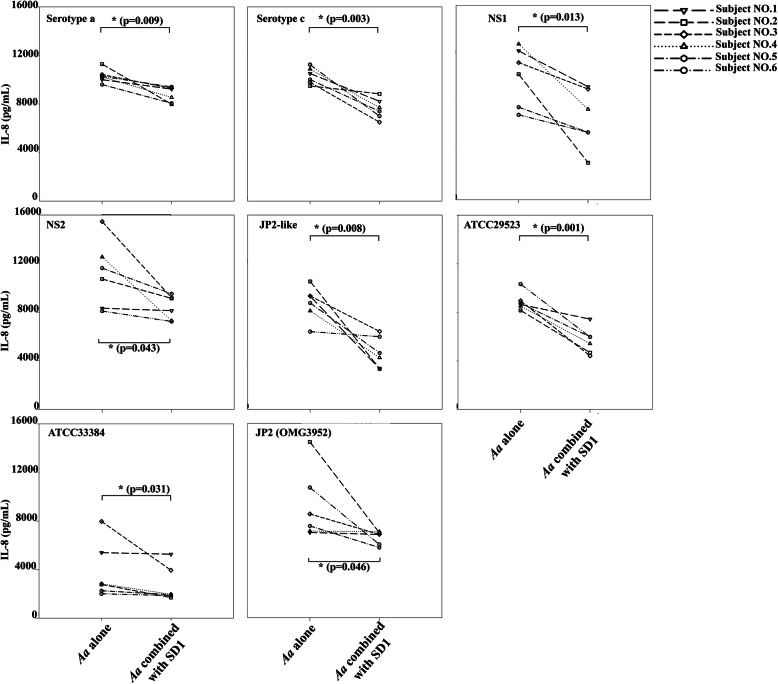
IL-8 secreted from PBMCs from six blood donors and exposed to CWEs (100 μg/mL) of eight strains of *A. actinomycetemcomitans* alone and in combination with *Lactobacillus paracasei* SD1. * Statistically significant cytotoxicity for the combinations compared to *A. actinomycetemcomitans* alone (Wilcoxon Signed-Rank Test; *p* < 0.05)

TNF-α (ranged 1256.6 to 51,103.8 pg/ml) showed the high levels when exposed to *A. actinomycetemcomitans* strains (Fig. 5) while IL-6 (ranged 10.34 to 631.9 pg/ml) and IL-8 (ranged 1009.9 to 15,480.6 pg/mL) were considerably lower than TNF-α (Fig. 6 and Fig. 7). After combination, *L. paracasei* SD1 reduced the secretion of IL-6, IL-8, and TNF-α significantly in all cell cultures also treated with the different *A. actinomycetemcomitans* strains (*p* < 0.05; Figs. 5, 6, 7). The reduced secretion of IL-6, IL-8, and TNF-α was 1.1–20.7, 1.1–3.4, and 1.1–11.5 folds, respectively. Strong individual variations were also seen here.

## Discussion

This study examined the effect of oral lactobacilli on the cytotoxicity and pro-inflammatory cytokine responses of PBMCs induced by eight different subtypes of *A. actinomycetemcomitans*. The main finding was that statistically significant reductions of the cytotoxicity and the pro-inflammatory cytokines, IL-1β, IL-6, IL-8, and TNF-α was demonstrated for oral *Lactobacillus* strains, and *L. paracasei* SD1 in particular, when combined with the *A. actinomycetemcomitans* subtypes although a significant variation between the blood donors was noticed. The study illustrates the high complexity in the interaction between oral bacteria and the host that exists even in vitro between bacterial species, species subtypes and components and between host (blood donor) cells such as PBMCs.

All *A. actinomycetemcomitans* and *Lactobacillus* strains showed a toxic effect and induced the release of cytokines from PBMCs in various degrees, although *A.actinomycetemcomitans* showed a generally and statistically significant higher toxic effect than the *Lactobacillus* strains. This finding was for both CWEs and supernatant indicating that various cell wall components as well as extracellular products from all bacteria, gram-negative as well as gram-positive, interfere with the host cells, in this case PBMCs. This means that the cytotoxicity as was measured in this study is of low specificity and basically not related to any particular component of the bacteria. This should be kept in mind for the complex in vivo situation with a dental biofilm with hundreds of different species in highly variable amounts. Thus, the effect of specific bacterial species, such as *A. actinomycetemcomitans* or lactobacilli, is generally unpredictable.

Not surprisingly the highly, according to the literature [6], toxic genotype JP2 of *A. actinomycetemcomitans* showed the highest toxic effect and significantly higher than the other *A. actinomycetemcomitans* subtypes. Similarly, the effect on IL-1β release was more pronounced for the JP2 clone, indicating that the effect on host cells may be specifically associated with leukotoxin production and with a potential co-stimulation of PBMCs by lipopolysaccharides (LPS). *A. actinomycetemcomitans* is a bacterial species associated with periodontitis due to its leukotoxin production, internalization into fibroblast cells, and immunomodulation [8, 9]. In a previous study it was shown that *A. actinomycetemcomitans* suppressed IL-8 mRNA expression and its function [9] as well as, this study revealed the potency of *A. actinomycetemcomitans* on stimulating PBMCs to secret IL-1β. Lipopolysaccharide (LPS) [9] and leukotoxin [14] of *A. actinomycetemcomitans* are immunodominant components that stimulate immune cell produced pro-inflammatory cytokines and chemokine, which is in accordance with the results of this study. *A. actinomycetemcomitans* CWEs and supernatant showed significantly higher cytotoxicity and IL-1β secretion by PBMCs compared to lactobacilli. The response to the highly toxic JP2 genotype (OMGS 3952) indicates that the leukotoxin plays a major role in the IL-1β release from PBMCs.

*A. actinomycetemcomitans* components other than the toxin appeared to be stronger regulators of pro-inflammatory cytokines such as IL-6, IL-8, TNF-α in the various experiments. The release of the cytokines IL-6 and IL-8 was low to moderate (Fig. 6 and Fig. 7) while the release of TNF-α was remarkably high for all *A. actinomycetemcomitans* subgroups (Fig. 5). The JP2 subtype (strain OMG3952) showed here the lowest response except for one of the blood donors (Fig. 5, OMGS 3952), indicating that other bacterial components than leukotoxin may be responsible for the TNF-α release.

Both supernatants and CWEs of oral lactobacilli reduced the cytotoxicity and pro-inflammatory cytokines of the PBMCs when combined with an adjusted amount of supernatant and CWEs of *A. acinomycetemcomitans* strains. In particular, the supernatant and CWEs of *L. paracasei* SD1 had a significant and consistent reduction on IL-1β, IL-6, IL-8, and TNF-α release on all subtypes of *A. acinomycetemcomitans*. The component responsible for the reducing effect in lactobacilli is not known. *Lactobacillus* strains have been recognized as beneficial bacteria along the gastrointestinal tract and suggested and used as a probiotic due to its both general inhibiting effect on other microorganisms e.g. by its acid production [10, 11] and specific effect on bacterial growth [15] and reducing effect on toxicity by bacteriocins [13]. The potential interactions in the regulation of the dental biofilm ecology are numerous and involve many different microbial species and the importance of specific interaction between lactobacilli and *A. actinomycetemcomitans,* as was shown in this in vitro study, can only at this stage be speculated. The reducing effect of the oral *Lactobacillus* strains on the toxicity of *A. actinomycetemcomitans* could be considered limited from this in vitro study in view of the rare colonization of lactobacilli in the periodontal pocket, however it is possible that certain strains may interact with the colonization and growth of *A. actinomycetemcomitans* in the early stages of periodontal disease.

The response was highly variable between the blood donors for both IL-1β and TNF-α and even non-responders were noticed as illustrated by subject 6 (Fig. 3) in whom IL-1β was not detected for any of the 8 subtypes of *A. actinomyctemcomitans*. This variation in host susceptibility for a putative periodontal pathogen such as *A. actinomycetemcomitans* and cell components and extracellular products from them illustrates the complexity of the interaction between the dental biofilm and host tissues in vivo and the variation in the periodontal disease susceptibility. It could be argued that the variation seen between the blood donors may be due to methodological errors in collecting, storage and preparing the buffy coats for the experiments. All buffy coats were ordered and prepared from blood samples the day before the experiment. At the day of the experiment, the cells from various buffy coats were treated in parallel and in same and standardized way. We therefore argue that methodological errors have only a minor impact in this study. Instead, this study show that substantial and inter-individual variation exists in cytokine release from the monocytes. Thus, there is a benefit of using buffy coats in studies to include this natural variation among different subjects, which is not possible when using cell lines.

## Conclusions

This study found a limited but statistically significant reducing effect of three oral *Lactobacillus* strains on the toxicity of eight *Aggregatibacter actinomycetemcomitans* strains. This in vitro study further demonstrates the complexity of the interactions among bacteria and also between bacteria and the host cells. The large variability among the target cells (*A. actinomycetemcomitans),* among the effector strains (lactobacilli), and among host cells (PBMCs from blood donors), illustrate this complexity. The most inhibited strains were the most toxic target strains (the JP2 clone), and *L. paracasei* SD1 had a more inhibiting effect than the other oral *Lactobacillus* strains.

## Methods

### Study design

The following factors were tested:

-The toxicity and cytokine release for the individual strains of *A. actinomyctemcomitans* and *Lactobacillus* spp.

- The toxicity and cytokine release of supernatant and cell wall extract (CWEs) of each strain respectively.

- The toxicity and cytokine release of a strain of *A. actinomycetemcomitans* alone and in combination with *Lactobacillus* strains.

- The diversity of cytokine release between blood donors.

### Bacterial strains

Five strains of *A. actinomycetemcomitans* were selected among clinical isolates from Thai adults with periodontitis and representing different subtypes (serotype a and c, a JP2 like serotype c, and 2 non-serotypable strains, NS1 and NS2, representing two different DGGE subtypes that have shown a high toxicity in a previous study [8]) were included as target strains for the study together with 3 reference strains representing serotype a (ATCC29523), serotype c (ATCC33384), and the JP2 clone serotype b (OMGS 3952). The latter stain was isolated from a young girl of Cap Verde Island with an advanced periodontitis [16]. Further, *Lactobacillus paracasei* SD1 and *L. rhamnosus* SD11 previously tested as oral probiotic strains [17, 18] and a reference strain *L. rhamnosus* ATCC53103 (LGG) were used as effector strains. The *A. actinomycetemcomitans* strains were cultured in brain heart infusion broth (BHI, Acumedia, Neogen, Lansing, Mich, USA) and the *Lactobacillus* strains in *Lactobacillus* selective medium (Acumedia, Neogen, Lansing, Mich, USA) and were incubated at 37 °C for 48 h under anaerobic conditions.

### Bacterial supernatant preparation

The concentrations of *A. actinomycetemcomitans* were adjusted by the optical dentistry at OD_600_ = 0.15 which corresponded to 10^8^ CFU/mL in BHI broth and were incubated at 37 °C for 48 h under anaerobic conditions. Supernatants were collected after centrifugation at 2800 g for 10 min and then, the pH was adjusted to pH 7.0. The supernatants were filtrated and stored at − 20 °C until further analysis. The cell pellets were kept for cell wall extraction (see below).

The supernatant of *Lactobacillus* strains was prepared in the same way as for *A. actinomycetemcomitans*, although the concentration of *Lactobacillus* cells was adjusted at OD_600_ = 0.20) corresponding to cell counts of 10^8^ CFU/mL.

### Bacterial cell wall preparation

Cell pellets were used to extract cell wall components by differential centrifugation, as previously described [9]. Briefly, bacterial cells were resuspended in PBS pH 7.0 in the presence of a proteinase inhibitor cocktail (1 tablet yields a 1 mM EDTA solution in 10 ml, Roche Molecular Biochemicals, Mannheim, Germany) and the cells were disrupted by sonication. Intact cells were removed by centrifugation at 2200 g for 10 min at 4 °C, whereas the cell wall extract (CWEs) were collected from the supernatant by centrifugation at 30,000 g for 20 min at 4 °C. The cell wall pellet was resuspended in 500 μL of PBS with pH 7.0, and the total protein concentration was determined by the Bradford assay [19].

### Human peripheral blood mononuclear cells (PBMCs) isolation

Buffy coats were prepared from fresh blood collected from six healthy blood donors at hospital blood bank of the Sahlgrenska University Hospital in Gothenburg, Sweden and used for the experiments within 24 h. The buffy coats were used after deidentification, and according to Swedish legislation section code 4§ 3p SFS 2003:460, no informed consent is needed. The PBMCs isolated from the buffy coat from each donor were used for each co-incubation with all tested bacteria to analyze cytotoxicity and cytokine secretions. The experiments were run in triplicates for each buffy coat.

Peripheral blood mononuclear cells **(**PBMCs) were isolated from buffy coats by centrifugation over Ficoll-Paque™ Plus density gradient (GE healthcare Bio-Sciences AB, Uppsala, Sweden). In brief, each buffy coat was diluted 1:1 with PBS pH 7.0 and layered on 3 ml of Ficoll-Paque and centrifuged at 400 g for 30 min at room temperature. The PBMCs were collected at the interphase and washed twice in PBS and finally resuspended in Dulbecco’s Modified Eagle Medium plus GlutaMAX™ (Gibco, Life Technologies, Paisley, UK) supplemented with 5% human serum (Sigma-Aldrich, St Louis, MO, USA) and 1% penicillin-streptomycin (Sigma-Aldrich), then the cells were counted using a hematocytometer.

### Bacterial exposure

The PBMCs were added to 96-well plates at 2 × 10^6^ cells/well and cultured in the presence of the various concentrations (50, 100, 200, 400, 800, 1000 μg/mL) of lactobacilli CWEs and tested for cytotoxicity and cytokine secretion in order to find a suitable concentration for the experiments. A concentration of 100 μg/mL of lactobacilli CWEs was used while the concentration of *A. actinomycetemcomitans* CWEs was obtained from our previous study [9]. For the supernatant of both strains, an undiluted supernatant was used throughout this study.

PBMCs were added to 96-well plates at 2 × 10^6^ cells/well and cultured in the presence of bacterial supernatants or 100 μg/mL CWEs of either *A. actinomycetemcomitans* or *Lactobacillus* strains alone or combinations for 2 h at 37 °C in 5% CO_2_ incubator*.* PBMCs cultured without bacterial components were used as controls. The cell-free culture supernatants were collected for IL-1β, IL-6, IL-8, and TNF-α determination and the PBMC cells were collected for the cytotoxicity test.

### Cytotoxicity and IL-1β determination

The cytotoxicity of *A. actinomycetemcomitans* or *Lactobacillus* strains, single or combinations, was determined using the modified trypan blue exclusion method [20]. The percentage of cytotoxicity was calculated by 100 – (surviving cells of the test/surviving cells of the control × 100).

IL-1β analysis of cell-free culture medium was performed using the DuoSet ELISA Development Kit (R&D Systems, Abingdon, UK) according to the manufacturer’s instructions. The plates were coated with capture antibody overnight. The cell culture supernatants were incubated with a cytokine-specific biotinylated detection antibody and marked with streptavidin-conjugated horseradish-peroxidase. After the addition of the substrate, the absorbance was measured using an ELISA microplate reader (Synergy 2, BioTek Instruments, Inc., Winooski, VT, USA) at 405 nm and compared to a standard curve in order to calculate the concentration presented as pg/mL.

### IL-6, IL-8, and TNF-α determination

The cytokines, IL-6, IL-8, and TNF-α, were measured using a custom-made multiplex assay (Bio-Plex Express Assay, Bio-Rad Laboratories, Hemel Hempstead, UK) based on Luminex xMAP technology according to the manufacturer’s instructions. Briefly, the standard was reconstituted and diluted in a fourfold dilution series. Antibody coupled capture beads were prepared and plated. After washing using a Bio-Plex Pro™ wash station (Biorad), diluted samples and standards were added to the beads in the wells. The plate was incubated on a shaker and after incubation and wash, detection antibodies were added to each well and after the streptavidin-phycoerythin solution (R&D Systems, Abingdon, UK) was added to the wells. In the last incubation step, beads were resuspended in assay buffer and the plate was read with a BioPlex 200 instrument equipped with BioManager analysis software (BioRad). The absolute concentrations of the samples were determined by comparing the bead colour and mean fluorescence intensity from each set of beads against an automatically optimized and manually verified standard curve. The cytokine concentration was presented as pg/mL.

### Statistical analysis

The results of bacterial CWEs and supernatant on PBMCs stimulation were compared using Mann–Whitney U Test while the results of SD1 combined with *A. actinomycetemcomitans* on PBMCs stimulation were analyzed with Wilcoxon Signed-Rank Test. A *p*-value < 0.05 was considered as statistically significant.

## Data Availability

All data generated or analyzed during this study are included in this published article.

## Abbreviations

BHI: Brain heart infusion
CWE: Cell wall extract
DGGE: Denaturated gradient gel electrophoresis
HGEC: Human gingival epithelial cells
IL: Interleukin
LPS: Lipopolysaccharide
NS: Non-serotypable
PBMCs: Peripheral blood mononuclear cells
TNF-α: Tumor necrotic factor-alfa
